# Innovating Computational Biology and Intelligent Medicine: ICIBM 2019 Special Issue

**DOI:** 10.3390/genes11040437

**Published:** 2020-04-17

**Authors:** Yan Guo, Xia Ning, Ewy Mathé, Kai Wang, Lang Li, Chi Zhang, Zhongming Zhao

**Affiliations:** 1 Department of Internal Medicine, Comprehensive Cancer Center, University of New Mexico, Albuquerque, NM 87131, USA; 2 Department of Biomedical Informatics, Ohio State University, Columbus, OH 43210, USA; Xia.Ning@osumc.edu (X.N.); Ewy.Mathe@osumc.edu (E.M.); Lang.Li@osumc.edu (L.L.); 3 Raymond G. Perelman Center for Cellular and Molecular Therapeutics, Children’s Hospital of Philadelphia, Philadelphia, PA 19104, USA; kaichop@gmail.com; 4 Department of Medical & Molecular Genetics, School of Medicine, Indiana University, Indianapolis, IN 46202, USA; czhang87@iu.edu; 5 Center for Precision Health, School of Biomedical Informatics, The University of Texas Health Science Center at Houston, Houston, TX 77030, USA

**Keywords:** cancer informatics, clustering algorithm, single cell omics, transcriptomic analysis and tools, statistical methods, microbiome research

## Abstract

The International Association for Intelligent Biology and Medicine (IAIBM) is a nonprofit organization that promotes intelligent biology and medical science. It hosts an annual International Conference on Intelligent Biology and Medicine (ICIBM), which was established in 2012. The ICIBM 2019 was held from 9 to 11 June 2019 in Columbus, Ohio, USA. Out of the 105 original research manuscripts submitted to the conference, 18 were selected for publication in a Special Issue in *Genes*. The topics of the selected manuscripts cover a wide range of current topics in biomedical research including cancer informatics, transcriptomic, computational algorithms, visualization and tools, deep learning, and microbiome research. In this editorial, we briefly introduce each of the manuscripts and discuss their contribution to the advance of science and technology.

## 1. Introduction

The International Conference on Intelligent Biology and Medicine (ICIBM 2019) was organized and hosted by the International Association for Intelligent Biology and Medicine (IAIBM) and the Department of Biomedical Informatics at Ohio State University from 9 to 11 June 2019 in Columbus, Ohio, USA. The detailed description of the conference and its organization and achievements is summarized in [1]. The conference attracted 164 researchers and over 100 manuscripts with broad scientific topics were submitted. Each manuscript went through ICIBM internal peer review by a minimum of two reviewers. Manuscripts selected for publication in the Special Issue in *Genes* were further reviewed by a minimum of two additional external reviewers. In the end, 18 manuscripts were selected for publication in the Special Issue, which covered the topics of cancer research, gene expression, single cell sequencing, novel computational algorithms, and microbiome research. In this editorial, we introduce the 18 selected research manuscripts.

## 2. Cancer Informatics

Chen et al. published “Computational Cancer Cell Models to Guide Precision Breast Cancer Medicine” [2]. In this work, the authors introduced an optimal two-layer decision system model for predicting drug sensitivity. The practical application of this model resides in precision or personalized medicine that attempts to address the bridge between conventional in vitro cancer cell models and clinical patient response to cancer drugs, which is still lacking. This model obtained an average accuracy of 90.8% using the simulated data. In conclusion, this two-layer model could be easily extended to multiple cancer types and it could help basic scientists who are seek optimal cancer cell models for an individual tumor while prioritizing clinical drugs’ recommendations in practice.

A pathway is a summary of a set of genes that can be connected via their biological process, regulation, mechanism. or phenomenon. Pathways of important function can be alternatively activated in cancer. Wang et al. published “Identification of Alternatively-Activated Pathways between Primary Breast Cancer and Liver Metastatic Cancer Using Microarray Data”, in which the authors proposed an alternatively-activated pathway mining method [3] based on microarray data, and identified three types of alternatively-activated pathways between primary breast cancer and breast liver metastatic cancer. There were three types of alternatively-activated pathways that were identified through their analysis as follows: active states of some gene pairs were inversed, some subpathways were only active in primary cancer or metastatic cancer, and some subpathways were alternatively-activated by different genes.

Circulating tumor DNA (ctDNA) has been found in the bloodstream which originated from cancerous cells. Research on ctDNA has been expanding over the last decade resulting in substantial advancement in the identification of single nucleotide variants from ctDNA. Copy number variation (CNV), which is also considered to be an important cancer biomarker, has been very difficult to detect from ctDNA due to the low amount and complex CNV features. Peng et al. published “CNV Detection from Circulating Tumor DNA in Late-Stage Non-Small Cell Lung Cancer Patients” to address the critical issue of CNV identification in ctDNA [4]. Their method could detect CNVs from a 150-gene panel using a very low amount of ctDNA. 

DNA methylation plays a variety of roles in cancer, including a critical role in the control of gene activity, which helps to convert gene expression in normal tissue to a cancerous pattern. Utilizing deep learning techniques, Liu et al. published “DNA Methylation Markers for Pan-Cancer Prediction by Deep Learning”, in which they studied the prognostic value of DNA methylation [5]. Using data from 27 cancer types covering 10,140 cancer samples and 3386 normal samples, the authors identified the following two categories of markers: 12 CpG markers and 13 promoter markers. The results were validated using cell-free DNA methylation data of 163 prostate cancer samples and they achieved excellent sensitivity and specificity. This study indicates that the identified biomarkers can be utilized in both cancer tissue and a cell-free setting.

Gene fusion describes hybrid genes that are formed from two independent genes. Gene fusion has been a common feature in cancer genomes and has served as a molecular target in therapeutic development. In Helm et al.’s “Gene Co-Expression Networks Restructured Gene Fusion in Rhabdomyosarcoma Cancers”, the authors studied gene fusion features in rhabdomyosarcoma [6]. In this study, the authors utilized co-expression network to study fusion between *FOXO1* and *PAX3/7* and observed substantial restructuring of co-expression networks related to fusion status and fusion type.

Tumor-infiltrating leukocytes (TILs) are immune cells surrounding tumor cells, and several studies have shown that TILs are potential survival predictors in several types of cancers including liver cancer, which is highly associated with a hepatitis virus. Hsiao et al. studied TIL abundance and compositions concerning hepatocellular carcinomas survival in their manuscript, entitled “Tumor-Infiltrating Leukocyte Composition and Prognostic Power in Hepatitis B- and Hepatitis C-Related Hepatocellular Carcinomas” [7]. The authors found that the total abundance of TILs was higher in non-tumor tissue regardless of the HCC subtype. Alternatively, the specific TILs associated with overall survival (OS) and recurrence-free survival (RFS) varied between subtypes.

Network and biomarker analyses have been heavily utilized for cancer research. Liu et al. combined the concept of both a network and biomarker approach in their paper entitled “Network as a Biomarker: A Novel Network-Based Sparse Bayesian Machine for Pathway-Driven Drug Response Prediction” [8]. The authors developed a network-based sparse Bayesian machine (NBSBM) approach, which attempted to use a network as a drug response biomarker. NBSBM made use of the information encoded in a disease-specific (differentially expressed) network to improve its prediction performance in problems with a reduced amount of training data and a very high-dimensional feature space. This method provides a disease-specific network-based drug sensitivity prediction approach and can uncover the potential mechanisms of the action of drugs by selecting the most predictive sub-networks from the disease-specific network.

In the paper entitled “Kinetic Modeling of DUSP Regulation in Herceptin-Resistant HER2-Positive Breast Cancer”, Buiga et al. focused on the analysis of dual-specificity phosphatases (DUSPs) in HER2-positive breast cancer [9], a highly aggressive subtype of breast cancer. The authors investigated whether inhibiting certain DUSPs resensitized Herceptin-resistant breast cancer cells to the drug by building kinetic models. The authors observed good concordance between their model and real tumor data. Their data shows that kinetic modeling of signaling pathways can generate predictions that assist experimental research in the identification of potential targets for cancer treatment.

## 3. Clustering Algorithm

Compelling evidence has shown that microRNAs (miRNAs) can regulate genes and be associated with various cancers through a post-transcriptional suppression regulation mechanism. The dysregulation of miRNA can substantially alter the landscape of the transcriptome level of messenger RNAs (mRNAs). Dai et al. published “Identifying Interaction Clusters for miRNA and mRNA Pairs in TCGA Network”, which describes a novel cluster scoring method to identify mRNA and miRNA interaction pairs [10]. Their analysis identified 54 significant clusters in 15 cancer types using the data from The Cancer Genome Atlas (TCGA) project. 

Cryogenic electron microscopy (cryoEM) is an electron microscopy technique applied on samples cooled to cryogenic temperatures and embedded in an environment of vitreous water. It is often used to study structural biology. The analysis of cryoEM often involves a clustering algorithm. Al-Azzawi et al. published “A Super-Clustering Approach for Fully Automated Single Particle Picking in Cryo-EM”, a manuscript that describes a newly developed fully automated super-clustering algorithm for single particle picking in cyroEM micrographs. The authors focused on identifying, detecting, and picking particles of the complex and irregular shapes in micrographs with extremely low signal-to-noise ratio [11].

## 4. Single Cell Omics 

While single cell sequencing has quickly emerged as a powerful technology for measuring DNA variants or transcriptome abundance at the single cell resolution, numerous challenges currently remain in this new field. One of the major shortcomings of single cell RNA-seq is the excessive zero counts, because only a small fraction of the transcripts sequenced in each cell. This problem can be alleviated by sequencing more, but it is at a high financial cost and also a lack of enough cells available for sequencing. The sparsity of the gene expression for each cell creates additional downstream analysis challenges such as cell type identification. Zand et al. introduced a network-based method, netImpute [12] to battle this problem in their manuscript, entitled “Network-Based Single-Cell RNA-Seq Data Imputation Enhances Cell Type Identification”. The netImpute method employs the random walk with the restart algorithm to adjust the gene expression level in a given cell by borrowing information from its neighbors in a gene co-expression network. Evaluation of seven real datasets showed that netImpute substantially enhanced cell type clustering accuracy and data visualization clarity. Mallik et al. published “Multi-Objective Optimized Fuzzy Clustering for Detecting Cell Clusters from Single-Cell Expression Profiles”, a study about cell clusters with a multi-objective optimization-based fuzzy clustering approach [13]. The authors demonstrated the approach in several real datasets and showed the usefulness of the method in detecting cell clusters from single cell RNA-seq data. 

## 5. Transcriptomic Analysis and Tools

In the era of big data, data visualization tools are essential for analyzing massive amounts of information and making data-driven decision. This is no difference in transcriptomic data analysis. Al-Ouran et al. published “A Portal to Visualize Transcriptome Profiles in Mouse Models of Neurological Disorders”, in which they described a new web-based platform for visualizing mouse transcriptome data [14]. The web portal was developed to help with nominating the best mouse models for studying neurological diseases. The portal can be used to examine gene expression changes across multiple mouse model studies including Alzheimer’s disease, Parkinson’s disease, Huntington’s disease, Amyotrophic Lateral Sclerosis, Spinocerebellar ataxia, and aging-related diseases.

Non-coding RNA has been the focus of many research studies over the last decade. Porto et al. published “Long Non-Coding RNA Expression Levels Modulate Cell-Type-Specific Splicing Patterns by Altering Their Interaction Landscape with RNA-Binding Proteins”, a study [15] in which the authors explored the role of lncRNAs in modulating alternative splicing and their impact on downstream protein–RNA interaction networks. On the basis of the analysis results, the authors proposed that such lncRNA sponges could extensively rewire post-transcriptional gene regulatory networks by altering the protein–RNA interaction landscape in a cell-type-specific manner.

A circadian rhythm is a natural internal process that regulates the sleep-wake cycle. While the canonical circadian clock genes and their regulatory mechanisms appear highly conserved, the evolution of clock gene families is still unclear due to several rounds of whole genome duplication in vertebrates. Sun et al. studied circadian clock genes in spotted gar, a non-teleost ray-finned fish, and published their findings in the manuscript “The Molecular Evolution of Circadian Clock Genes in Spotted Gar (*Lepisosteus oculatus*)” [16]. Phylogenetic analysis showed that nine of these 11 spotted gar circadian clock gene families have the same number of genes as humans, while the members of the *nfil3* and *cry* families are different between spotted gar and humans. These findings help decipher the repertoires of the spotted gar’s circadian system and shed light on how the vertebrate circadian clock systems have evolved.

## 6. Statistical Methods

In data science, a large dataset is often assembled from multiple smaller datasets with heterogeneity. The missing variable has become a common problem when combining datasets, which poses a major challenge for downstream analysis. Bartlett et al. published “Forming Big Datasets through Latent Class Concatenation of Imperfectly Matched Databases Features”, to address this problem [17]. The authors introduced ROSETTA, a statistical method to address missing variables. It empirically derives a set of common latent trait metrics for each related measurement domain using a novel variation of factor analysis to ensure equivalence across the constituent datasets. The advantages of combining datasets this way are the simplicity, statistical power, and modeling flexibility of a single joint analysis of all the data.

Genotyping data has been aiding researchers for large genetic association studies for the last two decades. Imputation is an important preprocessing step for combining genotyping data or increasing coverage. Traditional genotype imputation methods are typically based on haplotype-clustering algorithms, hidden Markov models (HMMs), and statistical inference. Chen et al. described their new deep learning-based imputation method [18] in their manuscript “Sparse Convolutional Denoising Autoencoders for Genotype Imputation”. The authors proposed a deep learning model named a sparse convolutional denoising autoencoder to impute missing genotypes. Their method showed strong robustness and outperformed popular reference-free imputation methods.

## 7. Microbiome Research

The microbiome studies many microorganisms in a particular environment. Microbiome research has been greatly enhanced by the advancement of 16S rRNA high throughput sequencing. Liu et al. published “Changes in the Microbial Community Diversity of Oil Exploitation”, a study on microbiome in several offshore petroleum production sites [19]. The authors observed a decrease of microbial community richness and diversity in petroleum mining, and the microbial community structure was strongly affected. Their study revealed that the various microbiomes produced surfactants, transforming the biohazard and degrading hydrocarbon. Altering the microbiome growth condition by appropriate human intervention and taking advantage of natural microbial resources can further enhance oil recovery technology.

## 8. Conclusions

ICIBM is an annual international conference, which has been held every year since 2012 (except 2017). It promotes a highly interactive and friendly platform for both young and senior researchers to exchange their research, foster collaboration, as well as expand educational activities. Approximately one hundred and seventy researchers and trainees from around the world joined the 2019 conference and contributed to a rich conference program, which included four keynote lectures, four eminent scholar talks, five tutorials and workshops, twelve concurrent sessions, a poster session, and other conference activities. Among the 105 original research manuscripts, we selected 18 for the Special Issue after two rounds of peer reviews. These 18 manuscripts describe innovative, computational works in the field. We expect these manuscripts to promote further investigation in the same or similar topics, and lead to more research toward translational clinical applications. 
